# CARTAR: a comprehensive web tool for identifying potential targets in chimeric antigen receptor therapies using TCGA and GTEx data

**DOI:** 10.1093/bib/bbae326

**Published:** 2024-07-08

**Authors:** Miguel Hernandez-Gamarra, Alba Salgado-Roo, Eduardo Dominguez, Elena María Goiricelaya Seco, Sara Veiga-Rúa, Lucía F Pedrera-Garbayo, Ángel Carracedo, Catarina Allegue

**Affiliations:** Genomic Medicine Group, Center for Research in Molecular Medicine and Chronic Diseases (CiMUS), University of Santiago de Compostela, Av. Barcelona, 15706 A Coruña, Spain; C005, Instituto de Investigación Sanitaria de Santiago (IDIS), Travesía da Choupana, 15706 A Coruña, Spain; Genomic Medicine Group, Center for Research in Molecular Medicine and Chronic Diseases (CiMUS), University of Santiago de Compostela, Av. Barcelona, 15706 A Coruña, Spain; C005, Instituto de Investigación Sanitaria de Santiago (IDIS), Travesía da Choupana, 15706 A Coruña, Spain; C005, Instituto de Investigación Sanitaria de Santiago (IDIS), Travesía da Choupana, 15706 A Coruña, Spain; C005, Instituto de Investigación Sanitaria de Santiago (IDIS), Travesía da Choupana, 15706 A Coruña, Spain; Genomic Medicine Group, Center for Research in Molecular Medicine and Chronic Diseases (CiMUS), University of Santiago de Compostela, Av. Barcelona, 15706 A Coruña, Spain; C005, Instituto de Investigación Sanitaria de Santiago (IDIS), Travesía da Choupana, 15706 A Coruña, Spain; Genomic Medicine Group, Center for Research in Molecular Medicine and Chronic Diseases (CiMUS), University of Santiago de Compostela, Av. Barcelona, 15706 A Coruña, Spain; C005, Instituto de Investigación Sanitaria de Santiago (IDIS), Travesía da Choupana, 15706 A Coruña, Spain; Genomic Medicine Group, Center for Research in Molecular Medicine and Chronic Diseases (CiMUS), University of Santiago de Compostela, Av. Barcelona, 15706 A Coruña, Spain; C005, Instituto de Investigación Sanitaria de Santiago (IDIS), Travesía da Choupana, 15706 A Coruña, Spain; Fundación Pública Galega de Medicina Xenómica (FPGMX), Travesía da Choupana, 15706 A Coruña, Spain; CB06/07/0088, Center for Biomedical Network Research on Rare Diseases (CIBERER), Instituto de Salud Carlos III, Av. Monforte de Lemos, 28029 Madrid, Spain; Genomic Medicine Group, Center for Research in Molecular Medicine and Chronic Diseases (CiMUS), University of Santiago de Compostela, Av. Barcelona, 15706 A Coruña, Spain; C005, Instituto de Investigación Sanitaria de Santiago (IDIS), Travesía da Choupana, 15706 A Coruña, Spain; CB06/07/0088, Center for Biomedical Network Research on Rare Diseases (CIBERER), Instituto de Salud Carlos III, Av. Monforte de Lemos, 28029 Madrid, Spain

**Keywords:** CAR, target selection, immunotherapy, web tool, logic-gated

## Abstract

Chimeric antigen receptor (CAR) therapy has emerged as a ground-breaking advancement in cancer treatment, harnessing the power of engineered human immune cells to target and eliminate cancer cells. The escalating interest and investment in CAR therapy in recent years emphasize its profound significance in clinical research, positioning it as a rapidly expanding frontier in the field of personalized cancer therapies. A crucial step in CAR therapy design is choosing the right target as it determines the therapy’s effectiveness, safety and specificity against cancer cells, while sparing healthy tissues. Herein, we propose a suite of tools for the identification and analysis of potential CAR targets leveraging expression data from The Cancer Genome Atlas and Genotype-Tissue Expression Project, which are implemented in CARTAR website. These tools focus on pinpointing tumor-associated antigens, ensuring target selectivity and assessing specificity to avoid off-tumor toxicities and can be used to rationally designing dual CARs. In addition, candidate target expression can be explored in cancer cell lines using the expression data for the Cancer Cell Line Encyclopedia. To our best knowledge, CARTAR is the first website dedicated to the systematic search of suitable candidate targets for CAR therapy. CARTAR is publicly accessible at https://gmxenomica.github.io/CARTAR/.

## Graphical Abstract

**Graphical Abstract:**
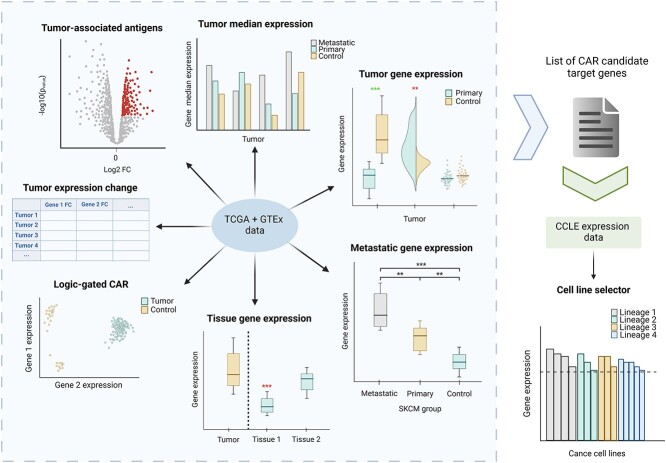

*In silico* identification and validation of potential immunotherapy targets located in cell plasma membrane.

## Introduction

Chimeric antigen receptor (CAR) modified cells represent a ground-breaking advancement in cancer immunotherapy, harnessing the patient’s immune system to selectively identify and eliminate cancer cells. While this precision medicine strategy has achieved remarkable clinical success in hematological malignancies [1, 2], prompting endeavors are still on the work to extend its application to solid tumors [3]. The key to successful CAR therapy lies in the careful selection of a suitable target, a challenge that currently lacks specific guidelines or criteria beyond the requisites of specificity and coverage [4]. This step is critical to ensure tumor regression while minimizing off-tumor toxicities.

CAR molecules target a variety of antigens on the surface of tumor cells, ranging from proteins to carbohydrates and glycolipids. The interaction between CAR and its targets triggers the immune synapse, culminating in contact-dependent cytotoxicity. Given that most tumor cells must be wiped out to ensure complete remission, ideal antigen targets should have high coverage on cancerous cells (e.g. CD19 against B-cell leukemia) [5, 6]. A second tenet of target selection is specificity to prevent CAR cells from causing harm to healthy organs. Therefore, an optimal target must exhibit both high coverage and specificity to achieve the required therapeutic effectiveness and safety for clinical use approval.

Yet, ideal targets are elusive, especially in solid tumors. In such cases, logic-gating CAR strategies offer a promising solution to overcome this hurdle. These strategies involve engaging two antigens to trigger effector cells activation upon binding to both antigens simultaneously (AND-gate); either one of the two antigens independently (OR-gate); to one antigen depending on the presence of the second (IF-BETTER-gate); or one antigen in the absence of the other (NOT-gate) [7, 8].

Currently, numerous valuable tools are available for gene expression visualization and analysis, such as cBioPortal [9], FireBrowse [10], GENT2 [11], GEPIA [12], TNMplot [13] and Xena [14]. The success of CAR therapies relies on meticulous target selection, requiring a comprehensive evaluation, and while these tools offer several useful features, they lack essential functionalities for CAR therapy target identification (Table 1).

**Table 1:** CARTAR comparison to similar tools.

Tool name	Data sets	Functionalities	Reference
CARTAR	TCGA, GTEx, CCLE	Differential analysis, identify tumor-associated antigens, compare tumor expression with that of healthy tissues, gene correlation among tumor and control samples, cancer cell lines gene expression	This paper
cBioPortal	TCGA, ICGC, TARGET, others	Differential analysis, gene correlation, survival analysis, mutation profile, structural variants, cohort profile, etc	[9]
FireBrowse	TCGA	Differential analysis, cohort profile	[10]
GENT2	NCBI GEO	Differential analysis, cancer subtype profiling, prognostic value, meta-survival analysis	[11]
GEPIA	TCGA, GTEx	Differential analysis, survival analysis, gene correlation, principal component analysis	[12]
TNMplot	NCBI GEO, GTEx, TCGA, TARGET	Differential analysis, gene signature analysis, correlation analysis, functional analysis	[13]
Xena	TCGA, GTEx	Differential analysis, survival analysis, subtype profiling, cohort profile	[14]

Note: TCGA: The Cancer Genome Atlas; GTEx: Genotype-Tissue Expression; CCLE: Cancer Cell Line Encyclopedia (DepMap Portal)’ ICGC: International Cancer Genome Consortium; TARGET: Therapeutically Applicable Research to Generate Effective Treatments; NCBI GEO: National Center for Biotechnology Information Gene Expression Omnibus.

Researchers in this field aim to identify genes located on the membrane that are highly expressed in tumor samples compared to controls. It is also important to verify that tumor expression levels are higher than those in healthy tissues to specifically target cancer cells while minimizing off-tumor toxicities. Furthermore, visualizing gene expression correlations—distinguishing between control and tumor samples—assists in designing logic-gated targeting strategies, and the knowledge of target expression in cancer cell lines can be used to select those of interest for testing the therapy.

To address these critical needs, we introduce CARTAR, a web-based tool that integrates all these functionalities. CARTAR enables the suitability assessment of candidate targets using RNA-Seq data from projects such as TCGA, GTEx and CCLE (DepMap), offering easy, rapid and customizable visualizations, which can be complemented with other tools, while also generating a downloadable table containing all processed data. Thus, CARTAR serves as a valuable resource for advancing the precision and efficacy of CAR therapy through informed target selection, filling a void in the bioinformatic field.

## Materials and methods

### Implementations

The freely available CARTAR website is built with Python 3.9.13 and Streamlit Community Cloud (https://streamlit.io/). Plots are generated with matplotlib (https://matplotlib.org/) and plotly (https://plotly.com/) libraries, while all tables are generated through the Streamlit dataframe function, allowing for data querying and selection. The website can be accessed from different browsers and devices without any login requirement.

#### Raw data

**Figure 1:**
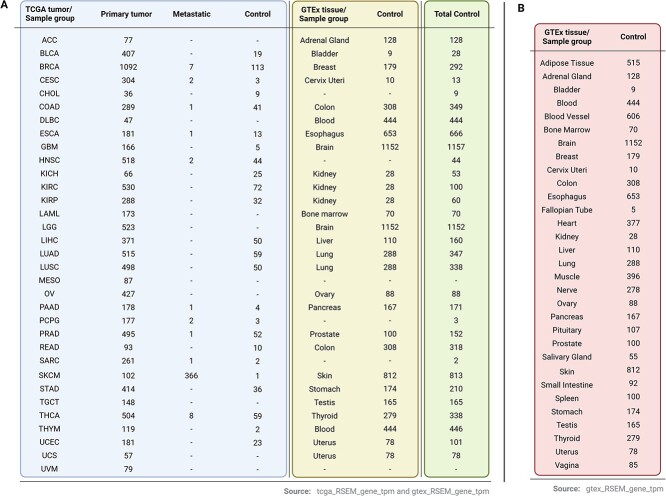
Sample classification. (A) Subdivision of TCGA tumors in sample group according to phenotype and inclusion of GTEx tissues included as control samples in each TCGA tumor. (B) Number of samples for each GTEx tissue.

The Cancer Genome Atlas (TCGA) project provides RNA-sequencing (RNA-Seq) data encompassing 9795 tumor samples, spanning 33 distinct cancer types, alongside 727 samples corresponding to adjacent normal tissue, referred here as control. To solve the inherent imbalance in sample sizes between tumor and control datasets, which compromise statistical significance in differential analyses, the inclusion of RNA-Seq data from 8295 control samples sourced from the Genotype-Tissue Expression (GTEx) project were included (Fig. 1). This addition ensures a comprehensive and representative dataset, mitigating the potential biases and fortifying the robustness of subsequent statistical analyses.

To guarantee uniformity and compatibility across the different sources, the UCSC Xena project (http://xena.ucsc.edu/) recomputed all raw expression data using a standardized pipeline. The recomputed raw RNA-Seq data from TCGA (tcga_RSEM_gene_tpm) and GTEx (gtex_RSEM_gene_tpm) were obtained through the UCSC Xena project, ensuring a harmonized and reliable foundation for subsequent analyses. This meticulous reprocessing not only enhances data consistency but also facilitates robust comparisons and integrations across genomic datasets avoiding batch effect. Phenotype data from both projects were downloaded from the UCSC Xena project for sample identification (TCGA_phenotype and GTEX_phenotype).

On the other hand, data from 1479 cancer cell lines were obtained, including metadata, by downloading the relevant information from the DepMap Public 23Q4 files (ExpressionProteinCodingGenes, DepMap23Q4Model_v2).

#### Data proccessing

**Figure 2:**
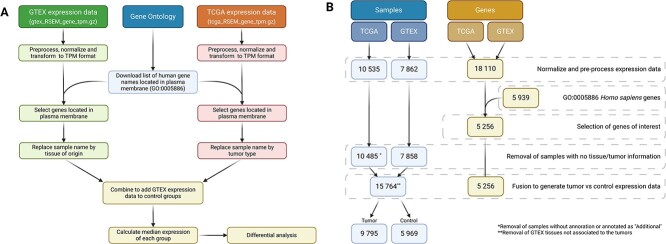
Raw data preprocessing. (A) Comprehensive bioinformatic pipeline used for raw data processing and web tool development. (B) Sample and gene filtering.

Raw data files underwent pre-processing using the scripts found in the Pre-processing folder of the CARTAR GitHub repository (https://gmxenomica.github.io/CARTAR/). This pre-processing step involves replacing the Ensemble ID with the corresponding gene symbol and replacing sample names with the respective cell line, tumor or tissue type information (Fig. 2). Additionally, the expression files from TCGA and GTEx were combined, retaining only the genes located in the plasma membrane (GO: 0005886), as CARs target tumor cells’ surface antigens. Finally, all necessary data were saved in dictionaries using the pickle library. $P$-values were calculated with the Mann–Whitney U test and multiple test correction was applied when required to calculate the adjusted $P$-value.

### Functionalities

The CARTAR website interface comprises eight primary features, each offering essential interactive functions, which include identifying tumor-associated antigens, conducting differential expression analysis, examining off-tumor gene expression, designing dual CARs and selecting cancer cell lines to evaluate candidate CAR therapies. Further details on these features are elaborated in the Results section. Additionally, a Help tab is available, offering a comprehensive user guide containing information about the website, descriptions of all available tools and guidance on interpreting the results.

CARTAR provides extensive customization options for data visualization, enabling users to tailor their experience. Users can choose the plot type, selecting from boxplots, violin plots or dot plots. Additionally, the website offers flexibility in expression scale, allowing users to opt for either transcripts per million (TPM) or log2(TPM+1). In the Tumor Expression Change tool, users can indicate the expression change between primary tumor and control samples using two distinct options: fold change (FC)—calculated as the median expression in primary tumor samples divided by the median expression in control samples—or log2(FC)—calculated as the median of log2(TPM+1) expression in primary tumor samples minus the median of log2(TPM+1) in control samples. These customization features enhance user control and facilitate precise exploration of expression patterns in the provided tools.

## Results

To offer practical guidance on how to use this website effectively and demonstrate its utility, a case study centered on identifying a target candidate for treating colorectal adenocarcinoma (COAD) is presented as an example.

### Tumor-associated antigens identification

**Figure 3:**
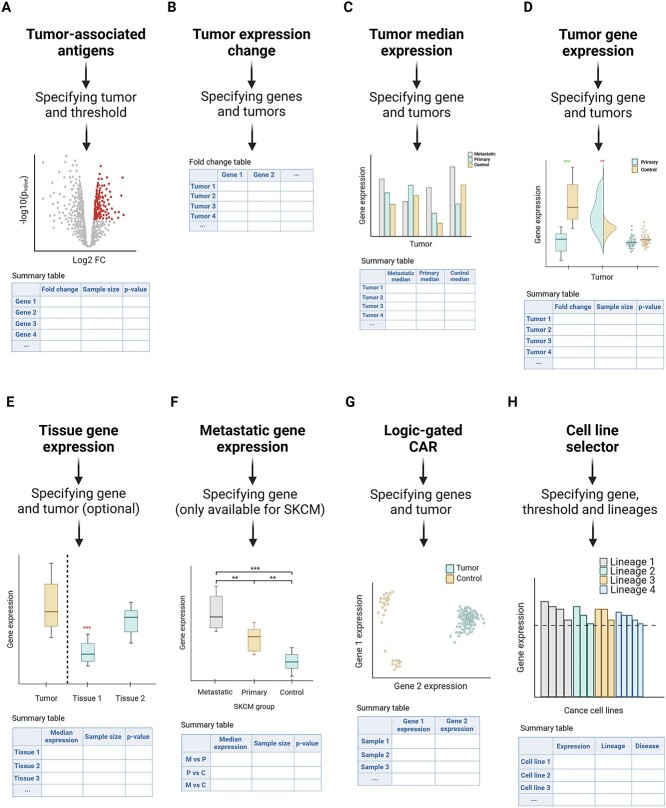
CARTAR tools workflow showing requested inputs and generated outputs. (A) Users can identify antigens overexpressed or underexpressed in primary tumor samples; (B) explore fold change expression values for candidate genes across selected tumors; (C) visualize median expression values of candidate genes across primary tumors, metastatic samples and control samples; (D) plot expression values of a specific gene across primary tumor and control samples of specified tumors; (E) and GTEx tissues; (F) plot gene expression values across metastatic, primary tumor and control samples of SKCM; (G) explore correlation between expression values of two genes for assessing their potential combination in logic-gated CAR therapies; and (H) identify cancer cell lines suitable for testing CAR activation and cytotoxicity against selected candidate genes.

This function allows users to discern antigens either overexpressed (tumor-associated antigens) or underexpressed in primary tumor samples in comparison to controls (Fig. 3A). Overexpressed genes, deemed primary CAR target candidates, are ideal for further exploration using CARTAR’s array of tools to validate their potential as therapeutic targets. Conversely, underexpressed genes hold potential for designing NOT-gated CAR therapies. Users can tailor their exploration by specifying the desired tumor and fold change, generating an interactive volcano plot for the 5,256 genes located in the plasma membrane. This plot highlights all significant genes (adjusted $P$-value<0.05) meeting the specified threshold, with gene name information accessible by hovering over the points. Additionally, a table is generated, providing comprehensive data on fold change, group medians, sample sizes, significance and $P$-values for genes meeting the threshold, serving as a crucial initial step in identifying potential targets aligned with user-defined specificity.

**Figure 4:**
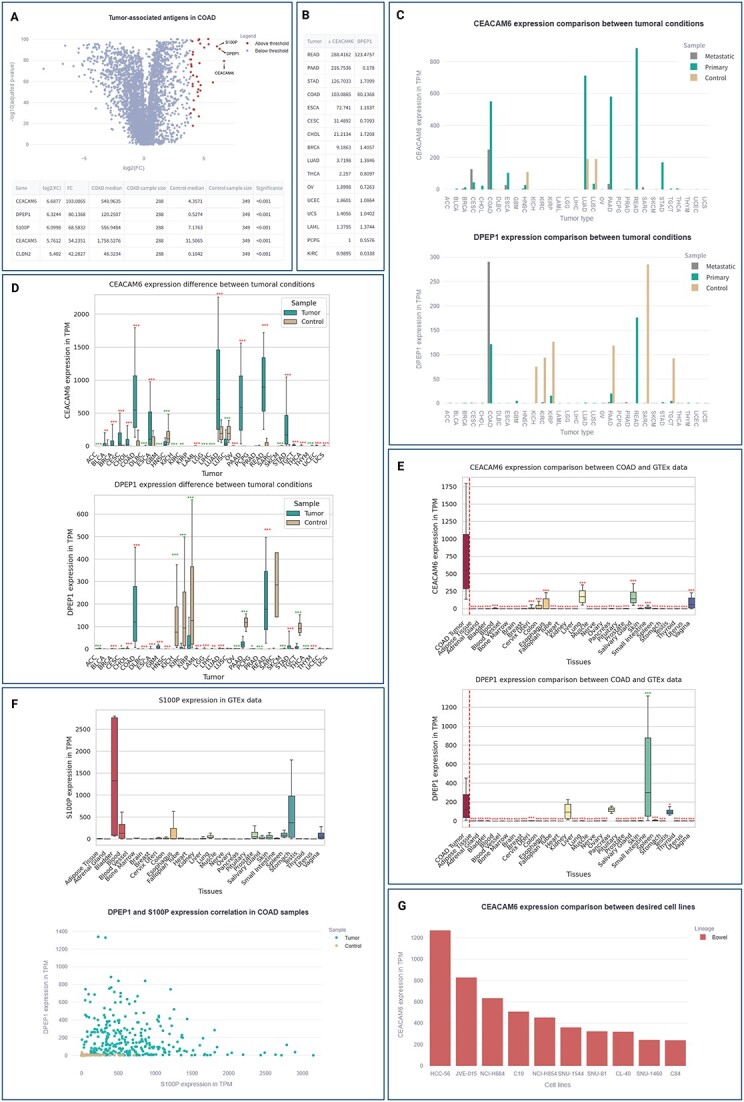
COAD cancer CAR target search case study. (A) Initial candidate search; (B) identification of other tumors where CEACAM6 and DPEP1 initial candidates can be used as targets; (C) visualization of CEACAM6 median expression (top) and DPEP1 (bottom) genes across the different conditions of tumoral groups; (D) boxplot of candidates expression in the tumoral groups to asses differential expression statistical significance and target selectivity; (E) boxplot comparing candidates expression of COAD tumoral samples with GTEx tissues to asses target selectivity; (F) S100P expression boxplot comparing COAD tumoral samples with GTEx tissues to asses target selectivity (top) and DPEP1/S100P expression correlation in COAD samples (bottom) to validate the design of an AND-gate target strategy; and (G) bowel lineage cancer cell lines with CEACAM6 gene expression higher than 200 TPM to select cell lines to test the proposed anti-CEACAM6 CAR.

For this case study, the objective was to identify surface targets with a minimum 15-fold increased expression in COAD tumoral samples compared to controls. The two candidate targets with the highest fold change (CEACAM6 and DPEP1) seem to show promising properties to become CAR targets, although they must be subjected to further evaluation (Fig. 4A). *CEACAM6* encodes a cell adhesion protein, with heightened expression linked to reduced patient survival [15]. Notably, other members of the same protein family have been previously identified as CAR targets for solid tumors [16, 17] supporting its suitability as a CAR target. On the other hand, *DPEP1* encodes a dipeptidase whose expression has already been reported to be increased in colorectal cancer samples [18], along with evidence suggesting a role in conferring drug resistance [19] underscoring its relevance in tumoral processes.

### Gene expression change across tumors

Users can select cancer groups of interest to explore fold change expression values for one or several candidate genes placed in the plasma membrane (Fig. 3B). Results are displayed on a table reporting the fold change—with log2(FC) option available—for each indicated gene across all selected tumors. This function facilitates the assessment of candidate target genes selectivity: whether it displays specific tumor-selectivity, or it can serve across several tumors.

Thus, Fig. 4B shows that CEACAM6 is a tumor-specific antigen not only in COAD but also in rectal, pancreatic and stomach adenocarcinomas, while DPEP1 is only overexpressed in rectal and colorectal adenocarcinomas. From this analysis, it becomes evident that CEACAM6 holds a distinct advantage, offering a broader potential application for treating adenocarcinomas within the digestive system.

### Gene median expression across tumors

This feature enables users to visualize median expression values of a candidate gene across primary tumors, metastatic samples—if available—and controls (Fig. 3C). An interactive bar plot and a table present median and sample size data for each group, offering insights into general gene expression across different tumors, including metastatic samples when available. It is worth noting that most metastatic samples have a very low sample size (Fig. 1A). These data also provide information of all tumors that have a profile suitable to be treated with the candidate target.

As depicted in Fig. 4C, *CEACAM6* exhibits high expression in tumors identified with the Gene Expression Change Across Tumors tool, with minimal expression in control tissue, except for lung (LUAD, LUSC) and head and neck samples (HNSC). However, this expression is approximately 200 TPM, one-third of the TPM expression value observed in tumoral groups. On the other hand, *DPEP1* presents a less favorable scenario, displaying elevated expression in kidney, pancreas, testicles and connective healthy tissue, raising concerns about potential off-tumor toxicity.

### Gene expression across tumors

Through this tool boxplots, violin plots or dot plots can be produced to analyze expression values of a specific gene across primary tumor and control samples of specified tumors (Fig. 3D). The plot is supported by a table containing relevant data, including median expression, sample size, fold change as log2(FC), significance and $P$-value. Unlike the Gene Median Expression tool, this function provides information of gene expression in all samples of specified tumors, except for the metastatic samples due to reduced sample size, which is not enough for statistical significance. Skin Cutaneous Melanoma (SKCM) is the only tumoral group with a sample size larger enough ($N$ = 366) to obtain statistical significance, and it has its own CARTAR tool.

Consistent with previous findings, the results presented in Fig. 4D support that, despite substantial variability among tumoral samples, *CEACAM6* is significantly overexpressed in all mentioned tumors. Conversely, *DPEP1* exhibits increased expression in healthy tissues, suggesting a lack of selectivity.

### Gene expression across GTEx tissues

This function (Fig. 3E) allows users to create boxplots, violin plots or dot plots for the expression values of a gene of interest across all GTEx tissues. Users can also decide to add primary tumor sample expression values of a specific cancer type to the plot, facilitating the expression comparison between primary tumor samples and each GTEx tissue. This is crucial for assessing the specificity of candidate target genes toward tumoral cells, ensuring non-tumoral tissues are recognized. Median expression values, sample sizes, log2(FC), significance of differential expression and $P$-values are also reported in table format.

Returning to the case study, results in Fig. 4E support the high specificity of CEACAM6 target expression in tumoral cells. However, DPEP1 exhibits high expression in an additional tissue not previously explored -the small intestine- where its expression surpasses tumoral sample levels. Consequently, DPEP1 does not appear to be an ideal CAR target at first glance. Nevertheless, logic-gating strategies involving dual-targeting CARs could be explored to enhance tissue selectivity, as it will be explored in following subheadings.

### Metastatic gene expression in SKCM

For SKCM tumors, users can explore the expression of a desired gene across all sample groups, including metastatic samples, through boxplots, violin plots and dot plots (Fig. 3F). The tool calculates and displays statistical significance of differential expression in the plots. Moreover, a table provides additional information, including median expression values, sample sizes for each group, log2(FC), statistical significance of differential expression between sample groups and $P$-values. Thus, additional information regarding metastatic SKCM can be compared with primary tumors and control samples.

### Gene correlation for logic gating CAR

This function enables users to explore the correlation between the expression values of two genes in primary tumor and control samples of a specified tumor (Fig. 3G). It assesses the potential of combining these genes for a logic-gated CAR therapy, enabling targeting of multiple antigens with a single CAR cell. Logic-gated CAR therapy is used to overcome target unspecificity and acquisition of therapy-induced resistance. Here, an interactive correlation plot and a table are provided, displaying the expression values of each gene across different samples. It allows to identify if any of the logic-gated strategies (OR, AND, NOT or IF-BETTER) can be applied to the selected targets.

To assess whether the DPEP1 target could be rescued by a logic-gated CAR, new antigens can be explored. One option is to identify an antigen expressed in colorectal tissue but not in kidney, testicles, small intestine and connective tissue to design an AND-gating CAR, requiring recognition of both antigens to be activated. S100P is proposed for this purpose as it appeared as the third antigen with higher fold change (Fig. 4A). Its expression was studied in the GTEx tissues (Fig. 4F top) to ensure *S100P* is not expressed in control tissues with high *DPEP1* expression levels. Although *S100P* is highly expressed in bladder, blood, esophagus and stomach cells, this is not problematic as *DPEP1* is not expressed in these tissues, and therefore the CAR would not be activated.

The expression correlation between both genes must be assessed with this tool to verify if the proposed gated-CAR is feasible. As shown in Fig. 4F (bottom), both target genes are highly expressed in tumoral samples, allowing their recognition and destruction by the proposed CAR therapy. In contrast, control colorectal samples exhibit little to no *DPEP1* expression, safeguarding them from the CAR cytotoxic activity.

### Cancer cell line selector

Users can also identify cancer cell lines to test CAR activation and cytotoxicity against selected candidates (Fig. 3H). After selecting the desired gene, cell lineage (optional) and threshold, an interactive barplots will display median expression values of the candidate gene for all cancer cell lines meeting the specified criteria. A table with additional information, including cell line catalog number, primary disease and subtype is also generated. This tool utilizes expression values from the Cancer Cell Line Encyclopedia (CCLE) and it assists in selecting cancer cell lines to test the cytotoxic activity of a CAR therapy targeted against a candidate gene, considering tumoral lineage a get gene expression levels or control cell lines based on low expression of the target gene.

To continue with the present example, colorectal cancer cell lines with high, medium and low CEACAM6 expression were identified to assess CAR activation and cytotoxicity at different target levels. The threshold can be established to show all bowel cancer cell lines with target expression over 200 TPM, based on gene expression in healthy tissues (Fig. 4G). Specifically, HCC-56 could be selected for high target expression (1270 TPM), NCI-H684 for medium expression (aligned with the COAD median expression of 635 TPM) and C84 for low expression (registered at 241 TPM).

## Discussion and conclusion

CARTAR has been designed to enable researchers without any computational programming skills to easily visualize and explore TCGA, GTEx and DepMap RNA-Seq expression data from genes located in plasma membrane. This tool focuses on the identification of candidate target genes for CAR and other immunotherapies and the suitability assessment of its coverage and specificity, differentiating itself from other tools of expression analysis. Besides, it allows to rationally design logic-gated CAR strategies and select cancer cell lines to test the CAR cells against a specific target.

The case study focused on COAD presented here demonstrates CARTAR’s utility, identifying CEACAM6 and DPEP1 as potential CAR targets due to their overexpression in primary tumor samples. These antigens are supported as adequate targets based on literature [20–22]. Notwithstanding, a logic-gated strategy with S100P is necessary for DPEP1 due to a lack of selectivity to avoid off tumor toxicities, mainly in kidney, pancreas and testis.

One of the limitations of the website is that targets are analyzed in terms of gene expression, missing information about post-translational modifications (PTM) such as phosphorylation, acetylation or glycosylation, that could be relevant for antigen recognition. Further developments on datasets related to PTM data addition can be implemented into CARTAR to enhance the potential of target identification. However, CARTAR will be updated to add new functionalities relevant for the field and solve these limitations.

In conclusion, CARTAR tool serves as a powerful ally in advancing the precision and efficacy of CAR therapy. By offering a systematic and data-driven approach to target selection and comprehensive analysis, CARTAR fills a critical gap in the personalized medicine and bioinformatic landscape. The showcased case study exemplifies CARTAR’s capabilities in guiding the selection of CAR targets, ensuring a thorough assessment of specificity, coverage and potential off-tumor toxicities. This web tool represents a significant stride towards informed decision-making in the evolving field of cancer immunotherapy.

**Key Points**

- CAR therapies target cancer cell-associated antigens located on the surface.
- We propose a novel bioinformatic methodology to identify cancer subtype-specific targets for CAR development.
- As an example, we identified candidate targets potentially suitable for colorectal cancer.
- The methodology is easy to use, straightforward and based on publicly available databases.

## Data Availability

CARTAR can be accessed at https://gmxenomica.github.io/CARTAR/. TCGA, GTEx (UCSC Xena) and CCLE (DepMap) expression raw data and metadata used can be found at tcga_RSEM_gene_tpm, TCGA_phenotype, gtex_RSEM_gene _tpm, GTEX_phenotype, DepMap23Q4OmicExpressionProtein CodingGenesTPMLogp1, DepMap23Q4Model_v2 links. The codes for data processing and website tools are accessible at https://github.com/GMXenomica/CARTAR/.

## Tumoral abbreviations

ACC: Adrenocortical carcinoma; BLCA: Bladder Urothelial Carcinoma; BRCA: Breast invasive carcinoma; CESC: Cervical squamous cell carcinoma and endocervical adenocarcinoma; CHOL: Cholangio carcinoma; COAD: Colon adenocarcinoma; DLBC: Lymphoid Neoplasm Diffuse Large B-cell Lymphoma; ESCA: Esophageal carcinoma; GBM: Glioblastoma multiforme; HNSC: Head and Neck squamous cell carcinoma; KICH: Kidney Chromophobe; KIRC: Kidney renal clear cell carcinoma; KIRP: Kidney renal papillary cell carcinoma; LAML: Acute Myeloid Leukemia; LGG: Brain Lower Grade Glioma; LIHC: Liver hepatocellular carcinoma; LUAD: Lung adenocarcinoma; LUSC: Lung squamous cell carcinoma; OV: Ovarian serous cystadenocarcinoma; PAAD: Pancreatic adenocarcinoma; PCPG: Pheochromocytoma and Paraganglioma; PRAD: Prostate adenocarcinoma; READ: Rectum adenocarcinoma; SARC: Sarcoma; SKCM: Skin Cutaneous Melanoma; STAD: Stomach adenocarcinoma; TGCT: Testicular Germ Cell Tumors; THCA: Thyroid carcinoma; THYM: Thymoma; UCEC: Uterine Corpus Endometrial Carcinoma; UCS: Uterine Carcinosarcoma.

## Author Biographies

**Miguel Hernandez-Gamarra** is a PhD student in the area of Health Sciences at the Universidade de Santiago de Compostela. His research interest is focused on designing CAR cell therapy, target selection and bioinformatics.

**Alba Salgado-Roo** is a PhD student in the area of Health Sciences at the Universidade de Santiago de Compostela. Her research interests lie in the field of proteomics and transcriptomics.

**Eduardo Dominguez, PhD** is a senior researcher at Instituto de Investigación Sanitaria de Santiago de Compostela (IDIS). His research interests include drug discovery and personalized medicine.

**Elena María Goiricelaya Seco, PhD** is a researcher at Instituto de Investigación Sanitaria de Santiago de Compostela (IDIS). She is specialized in RNASeq sample preparation and sequencing. She developed her PhD thesis in CAR based therapies (CAR19) against hematological neoplasms and applied to *in vitro* approach.

**Sara Veiga-Rúa** is a PhD student in the area of Health Sciences at the Universidade de Santiago de Compostela. Her research interest lies in the field of gene and cell therapy.

**Lucía F. Pedrera-Garbayo** is a PhD student in the area of Health Sciences at the Universidade de Santiago de Compostela. Her research interests lie in the field of transcriptomics and especially single cell RNAseq.

**Ángel Carracedo, MD, PhD** is a Full Professor of Faculty of Medicine, Universidade de Santiago de Compostela. He leads the genomic medicine group and numerous projects focused on the development of genomic medicine towards personalized diagnosis and treatment developments.

**Catarina Allegue, PhD** is a senior researcher at the Centre for research in Molecular Medicine and Chronic Diseases (CIMUS-Universidade de Santiago de Compostela). She leads the Disease Modeling and Advanced Therapy Developments including gene and cell therapies such as CAR-based therapies at the Genomic Medicine Group.
